# Assessment of the platelet-derived growth factor receptor alpha antibody olaratumab in a panel of patient-derived soft tissue sarcoma xenografts

**DOI:** 10.1186/s12885-019-5872-1

**Published:** 2019-07-22

**Authors:** Jasmien Cornillie, Agnieszka Wozniak, Britt Van Renterghem, Nathalie Van Winkel, Jasmien Wellens, Yemarshet K. Gebreyohannes, Maria Debiec-Rychter, Raf Sciot, Daphne Hompes, Patrick Schöffski

**Affiliations:** 1 Laboratory of Experimental Oncology, Department of Oncology and Department of General Medical Oncology, Leuven Cancer Institute, KU Leuven and University Hospitals Leuven, Leuven, Belgium; 2 Department of Human Genetics, KU Leuven and University Hospitals Leuven, Leuven, Belgium; 3 Department of Pathology, KU Leuven and University Hospitals Leuven, Leuven, Belgium; 4 Department of Surgical Oncology, KU Leuven and University Hospitals Leuven, Leuven, Belgium

**Keywords:** Olaratumab, Platelet-derived growth factor receptor alpha (PDGFRA), Soft tissue sarcoma, Patient-derived xenograft

## Abstract

**Background:**

Soft tissue sarcoma (STS) comprises a family of rare, heterogeneous tumors of mesenchymal origin. Single-agent doxorubicin remains the first-line standard-of-care treatment for advanced and inoperable STS, but response rates are only around 15%. In 2016, phase Ib/II clinical trial results reported an overall survival benefit of 11.8 months when combining doxorubicin and the platelet-derived growth factor receptor alpha (PDGFRA)-directed antibody olaratumab compared to doxorubicin alone, without providing a scientific rationale for such unprecedented therapeutic effect. We decided to evaluate the efficacy of olaratumab in a panel of STS patient-derived xenografts (PDX).

**Methods:**

NMRI *nu/nu* mice were bilaterally transplanted with tumor tissue of patient-derived xenograft models expressing PDGFRA, including models of leiomyosarcoma (UZLX-STS22), malignant peripheral nerve sheath tumor (UZLX-STS39), myxofibrosarcoma (UZLX-STS59) and undifferentiated pleomorphic sarcoma (UZLX-STS84). Mice were randomly divided into four different treatment groups: (1) control, (2) doxorubicin (3 mg/kg once weekly), (3) anti-PDGFRA [olaratumab (60 mg/kg twice weekly) + mouse anti-PDGFRA antibody 1E10 (20 mg/kg twice weekly)] and (4) the combination of doxorubicin and anti-PDGFRA (same dose/schedule as in the single treatment arms). Tumor volume, histopathology and Western blotting were used to assess treatment efficacy.

**Results:**

Anti-PDGFRA treatment as a single agent did not reduce tumor growth and did not result in significant anti-proliferative or pro-apoptotic activity. Combining doxorubicin and anti-PDGFRA did not reduce tumor burden, though a mild inhibition of proliferation was observed in UZLX-STS39 and -STS59. A pro-apoptotic effect was observed in all models except UZLX-STS22. Antitumor effects on histology were not significantly different comparing doxorubicin and the combination treatment. Moreover, anti-PDGFRA treatment, both as a single agent as well as combined with doxorubicin, did not result in inhibition of the downstream MAPK and PI3K/AKT signaling pathways.

**Conclusions:**

We were not able to demonstrate significant antitumor effects of anti-PDGFRA treatment in selected STS PDX models, neither alone nor in combination with doxorubicin. This is in line with the very recent results of the phase III clinical trial NCT02451943 ANNOUNCE, which did not confirm the clinical benefit of olaratumab in combination with doxorubicin over single agent doxorubicin.

**Electronic supplementary material:**

The online version of this article (10.1186/s12885-019-5872-1) contains supplementary material, which is available to authorized users.

## Background

Soft tissue sarcoma (STS) represents a diverse group of rare tumors of mesenchymal origin, accounting for about 1% of all adult malignancies [1]. The latest World Health Organization (WHO) classification defines more than 70 different histological subtypes of STS [2]. Unlike most epithelial cancers, the therapeutic landscape of advanced STS has not considerably evolved over the past decades. In the late 1970s, doxorubicin was the first cytotoxic agent showing a meaningful response rate in the setting of advanced STS and the drug remains the first line treatment of choice until today [3]. The combination of doxorubicin with ifosfamide increases response rates, but at the expense of a higher toxicity and without an overall survival (OS) benefit [4]. Second and later treatment lines comprise a variety of regimens such as dacarbazine, gemcitabine, trabectedin, pazopanib and eribulin [5–8], but high-level evidence to guide treatment beyond first line is lacking for the majority of histological subtypes, with the exception of leiomyo- and liposarcomas. Additionally, promising results of phase II studies are often not confirmed in the heterogeneous STS patient cohorts of subsequent phase III clinical trials as a number of them failed to improve the standard of care in the past years [9–11]. Taken together, treatment options for patients with advanced STS remain limited and prognosis for this patient population continues to be disappointingly low, with 5-year survival rates in the metastatic setting in the range of only 15% [1].

In view of the critical need for new active drugs for patients with this disease, the European Medicines Agency (EMA) and the U.S. Food and Drug Administration (FDA) recently granted accelerated approval to olaratumab for the treatment of advanced STS. Olaratumab is a human immunoglobulin G subclass 1 (IgG1) monoclonal antibody that specifically binds and inhibits the platelet-derived growth factor receptor alpha (PDGFRA). The platelet-derived growth factor (PDGF) family consist of 5 dimeric isoforms: the homodimers PDGF-AA, PDGF-BB, PDGF-CC, PDGF-DD and the heterodimer PDGF-AB. These isoforms exert their function through binding and activation of the tyrosine kinase receptors PDGFRA and platelet-derived growth factor receptor beta (PDGFRB). All PDGF isoforms, except for PDGF-DD, are able to induce PDGFRA dimerization, activation and autophosphorylation of the tyrosine kinase domains [12] and subsequent activation of the downstream phosphoinositide 3-kinase (PI3K) and mitogen-activated protein kinase (MAPK) pathways [13]. Additionally, binding of PDGF to its receptor can induce transactivation of the epidermal growth factor receptor (EGFR) [14]. PDGFRA is expressed in multiple tumor types including various types of sarcoma, both in tumor and stromal cells [12]. The receptor has a role in autocrine growth stimulation of the tumor cells, and in the recruitment of vascular endothelial growth factor (VEGF)-producing stromal fibroblasts, that have a critical role in tumor growth and angiogenesis [15, 16]. Additionally, PDGFRA expression has been associated with more aggressive tumor phenotypes and increased metastatic potential [17]. Therefore, olaratumab could theoretically have both a direct cytostatic effect by inhibiting tumor cell growth, as well as an indirect effect by reducing reactive stromal cells and inhibiting neo-angiogenesis. Indeed, olaratumab as single agent significantly delayed tumor growth in osteosarcoma and malignant rhabdoid tumor xenografts; combination of olaratumab with doxorubicin or cisplatin resulted in disease stabilization in osteosarcoma xenografts [18].

In the phase Ib/II clinical trial leading to accelerated approval of olaratumab, doxorubicin-naïve patients with advanced STS were randomly assigned to either doxorubicin monotherapy with a maximum of eight cycles of 75 mg/m^2^ doxorubicin on day 1 or to doxorubicin (same schedule as in the single treatment arm) in combination with 15 mg/kg olaratumab on days 1 and 8, with the option to continue olaratumab until disease progression [19]. The primary endpoint of the phase II part of the study was progression-free survival (PFS). This endpoint was not met, as only a non-significant PFS increase from 4.1 months in the doxorubicin monotherapy arm to 6.6 months in the combination group was observed. However, an unprecedented survival benefit of 11.8 months was achieved in the combination treatment arm compared to doxorubicin monotherapy. Interestingly, this gain in life expectancy could not be explained by the antitumor efficacy of the combination treatment, as objective response rates (ORR) did not differ between both treatment arms [19]. The results of a large, placebo-controlled ANNOUNCE phase III trial (www.clinicaltrials.gov: NCT02451943), aiming at providing confirmatory evidence for the activity of olaratumab in combination with doxorubicin and cardioprotective agents, are eagerly expected.

In this study, we explore the in vivo efficacy of olaratumab in patient-derived xenograft (PDX) models of different STS subtypes, aiming at a better understanding of the biological changes and antitumor effects upon PDGFRA-targeted treatment in STS. PDX models are established by directly transplanting human tumor tissue into immunodeficient mice and have shown to sustain the histological and molecular features of the original patients’ tumor tissue over time [20]. Therefore, PDX models are considered to more reliably mimic the human tumor biology compared to in vitro research or cell-line derived xenograft models.

## Methods

### Patient-derived STS xenograft models

STS xenografts were established in the Laboratory of Experimental Oncology, KU Leuven, Belgium, by bilateral subcutaneous transplantation of human tumor tissue in adult (10–12 weeks old) female, partially immunodeficient NMRI *nu/nu* mice (Janvier Laboratories), as previously described [21, 22]. All patients underwent surgery in the Department of Surgical Oncology or Orthopedic Surgery, University Hospitals Leuven (Belgium) and gave written informed consent for using the tissue to create PDX models and for the subsequent use of these models in translational research projects. Collection of tissue for xenografting was approved by the Medical Ethics Committee of the University Hospitals Leuven (S53483).

For the present study, we established PDX models derived from donor patients with different histological STS subtypes who all developed metastatic spread during the course of their disease. A PDX model was considered established after observing stable histological and molecular features for at least two subsequent passages in mice. The in vivo efficacy of olaratumab was tested in four PDX models that were selected based upon PDGFRA immunopositivity, assessed by Eli Lilly and Company, Indianapolis, United States, on tissue slides from all PDX models available in our laboratory at that time (*n* = 24). Based on these results, we used leiomyosarcoma (LMS) [UZLX-STS22 passage 18 (p.18)], malignant peripheral nerve sheath tumor (MPNST) (UZLX-STS39p.15), myxofibrosarcoma (UZLX-STS59p.19) and undifferentiated pleomorphic sarcoma (UPS) (UZLX-STS84p.10) xenografts for the experiments. Demographics and treatment history of the donor patients are presented in Table 1. All patients were chemotherapy-naïve at the time of xenografting, but all but one received systemic therapy after tissue collection.

**Table 1:** Demographics and treatment history of the STS PDX donor patients

PDX model	Gender	Age range at diagnosis (years)	STS subtype	Primary tumor location	Metastatic disease at time of engraftment	Metastatic disease after engraftment	Treatment response (after xenografting)
UZLX-STS22	Male	50–60	Leiomyosarcoma	Quadriceps muscle	No	Yes	PD under doxorubicin + evofosfamide PD under trabectedin
UZLX-STS39	Female	60–70	Malignant peripheral nerve sheath tumor	Abdominal wall	No	Yes	Refusal of chemotherapy
UZLX-STS59	Male	50–60	Myxofibrosarcoma	Adductor muscle	Yes	Yes	PD under doxorubicin PD under ifosfamide PD under pazopanib PD under trabectedin
UZLX-STS84	Female	70–80	Undifferentiated pleomorphic sarcoma	Adductor muscle	No	Yes	PD under pazopanib

*PD* progressive disease, *PDX* patient-derived xenograft, *STS* soft tissue sarcoma

### Drugs and reagents

Anti-PDGFRA mixture [olaratumab plus mouse anti-PDGFRA (1E10)] and human IgG control antibodies, all dissolved in sterile phosphate-buffered saline, were provided by Eli Lilly and Company. Doxorubicin hydrochloride was purchased from Sigma-Aldrich and was dissolved in sterile 0.9% sodium chloride solution.

The following antibodies were used for immunohistochemistry (IHC): alpha-smooth muscle actin (α-SMA) and S100 (DAKO), cleaved poly (ADP-ribose) polymerase (cleaved PARP) and human leucocyte antigen A (HLA-A) (Abcam), phospho-histone H3 (pHH3) and PDGFRA (Cell Signaling Technology), and murine CD31 (Dianova). All sections were incubated with secondary antibody-horseradish peroxidase polymer conjugate (Envision+ System-HRP, DAKO), except for cleaved PARP [SignalStain Boost IHC Detection Reagent (Cell Signaling Technology)] and CD31 [biotinylated secondary antibody (Vector Laboratories)]. Subsequently, stainings were developed using diaminobenzidine (DAB; DAKO), followed by hematoxylin counterstaining (VWR). The immunohistochemical assessment of PDGFRA was performed with PDGFRA rabbit monocloncal antibody (Cell Signaling Technology clone D13C6), the same that was used for PDX models screening by Eli Lilly and Company.

The following antibodies were used for Western blotting: PDGFRA, phospho-PDGFRA Tyr 849 / phospho-PDGFRB Tyr 857 (pPDGFRA/B), PDGFRB, phospho-PDGFRB Tyr771 (pPDGFRB), EGFR, phospho-EGFR Tyr1068 (pEGFR), AKT, phospho-AKT Ser473 (pAKT), ribosomal protein S6 (RPS6), phospho-RPS6 Ser235/236 (pRPS6), eukaryotic translation initiation factor 4E-binding protein 1 (4E-BP1), phospho-4E-BP1 Ser65 (p4E-BP1), mitogen-activated protein kinase (MAPK), phospho-MAPK Thr202/Tyr204 (pMAPK), histone H3, pHH3 and alpha-tubulin (all Cell Signaling Technology). MG-63 human osteosarcoma cell line stimulated with PDGF-BB, provided by Eli Lilly and Company, was used as a positive control for (p)PDGFRA/B. Human embryonic kidney 293 cells (HEK293; ATCC) was used as a positive control for (p)EGFR. Specific bands were visualized using the Western Lightning ECL detection kit (Perkin Elmer).

### Study design

A total of 95 adult (10–12 weeks old), female, partially immunodeficient NMRI *nu/nu* mice were engrafted bilaterally with UZLX-STS22p.18 (*n* = 24), UZLX-STS39p.15 (n = 24), UZLX-STS59p.19 (n = 24) and UZLX-STS84p.10 (*n* = 23) tumor tissue. Eleven out of the 190 engrafted tumor specimens did not grow, however, after randomization at least 7 tumors were included in each treatment group. A detailed description of the number of mice/tumors included in the study can be found in Additional file 2: Table S1. Two tumors from the same mouse were considered as independent events and were therefore analyzed separately. When tumors reached an average volume of around 250 mm^3^, mice were randomly assigned to one of four treatment groups: (1) a control group treated with human IgG control antibody [80 mg/kg intraperitoneally (i.p.) twice a week (BIW)], (2) doxorubicin [3 mg/kg i.p. once a week (QW)], (3) anti-PDGFRA mixture of olaratumab (60 mg/kg i.p. BIW) and mouse anti-PDGFRA antibody 1E10 (20 mg/kg i.p. BIW) and (4) combination of doxorubicin and the anti-PDGFRA mixture (same dose and schedule as in the single treatment arms; administered on the same day with a 2 h difference between both administrations). After several dose titration experiments with various concentrations and dosing frequencies, 3 mg/kg i.p. QW was considered the maximum tolerated dose of doxorubicin in our NMRI *nu/nu* mice and under our experimental conditions. The dose of olaratumab was based on previously published in vivo work with this drug, showing antitumoral efficacy in osteosarcoma xenografts [18]. In the experimental treatment arm both human and murine anti-PDGFRA antibody was administered as anti-PDGFRA mixture, in order to evaluate the effect of PDGFRA inhibition not only in the tumor cells, but also in the tumor stroma, that is known to be murine after several passages [22]. Mice were housed in individually ventilated cages (4 mice per cage) in a conventional mouse facility with wood fiber bedding material and *ad libitum* access to water and food. Tumor volume, histopathology and Western blotting were used to assess treatment efficacy. Tumor volume was assessed three times a week with 3D caliper measurement and normalized against baseline values. Body weight and general wellbeing of mice were followed daily. Animals in the different treatment arms were assessed and treated in a random order. Criteria to euthanize animals before the planned end of the experiment were (a) 15% body weight loss in a short time or 20% body weight loss since the beginning of the experiment, (b) excessive tumor growth and (c) serious symptoms due to tumor growth and/or treatment. After two weeks of treatment, mice were routinely euthanized. Half of each tumor was collected in 4% buffered formaldehyde for further histological assessment, the other half was snap-frozen in liquid nitrogen for molecular analysis. Euthanasia was performed using an overdose of pentobarbital, followed by cervical dislocation once mice were fully sedated. All animal experiments were conducted in accordance with Belgian law and approved by the Animal Ethics Committee, KU Leuven (project P175/2015).

### Histological assessment

Formaldehyde-fixed tumor specimens were embedded in paraffin and cut into 4-μm sections for hematoxylin and eosin (H&E) staining and IHC analysis. Mitotic and apoptotic activity were assessed on H&E-stained sections by counting mitotic and apoptotic cells in 10 high power fields (HPFs) at 400-fold magnification (0.55-mm field diameter). pHH3 and cleaved PARP immunostainings, as markers for proliferative and apoptotic activity respectively, were assessed by counting the number of positive tumor cells in 10 HPFs. Microvessel density (MVD) was calculated as the number of CD31-positive vessels in 5 HPFs at 200-fold magnification (1.1-mm field diameter) to assess vascular effects of PDGFRA inhibition. Histological analysis was performed blinded to the different treatment arms, using a BX43 light microscope (Olympus). Images were analyzed using the CellSens Dimension imaging software (version 1.16, Olympus). Original patient samples used to generate the PDX models were analyzed by a reference pathologist, using a DM 2000 LED microscope (Leica Microsystems).

### Western blotting

Tumor lysates were prepared from snap-frozen tumor samples as previously described [23], separated on Nu-Page 4–12% Bis-Tris gels (Life Technologies) and blotted to polyvinylidene difluoride (PVDF) membranes (Bio-Rad). Levels of chemiluminescence were captured using the FUJI-LAS mini 3000 system (Fujifilm). Densitometric analysis was performed to semi-quantify the protein expression, using the Advanced Image Data Analyzer (AIDA) software (version 4.15, Raytest).

### Statistical analysis

The Wilcoxon matched pairs test (WMP) was used to compare tumor volume at the end of the in vivo experiment vs. baseline. To compare tumor volume and histological assessment between different treatment groups, the Mann-Whitney U test (MWU) was applied. A *p*-value below 0.05 was considered statistically significant. The statistical analysis was performed with the GraphPad Prism software (version 7.01, GraphPad Software).

## Results

### Histological characterization of the established STS PDX models

**Fig. 1:**
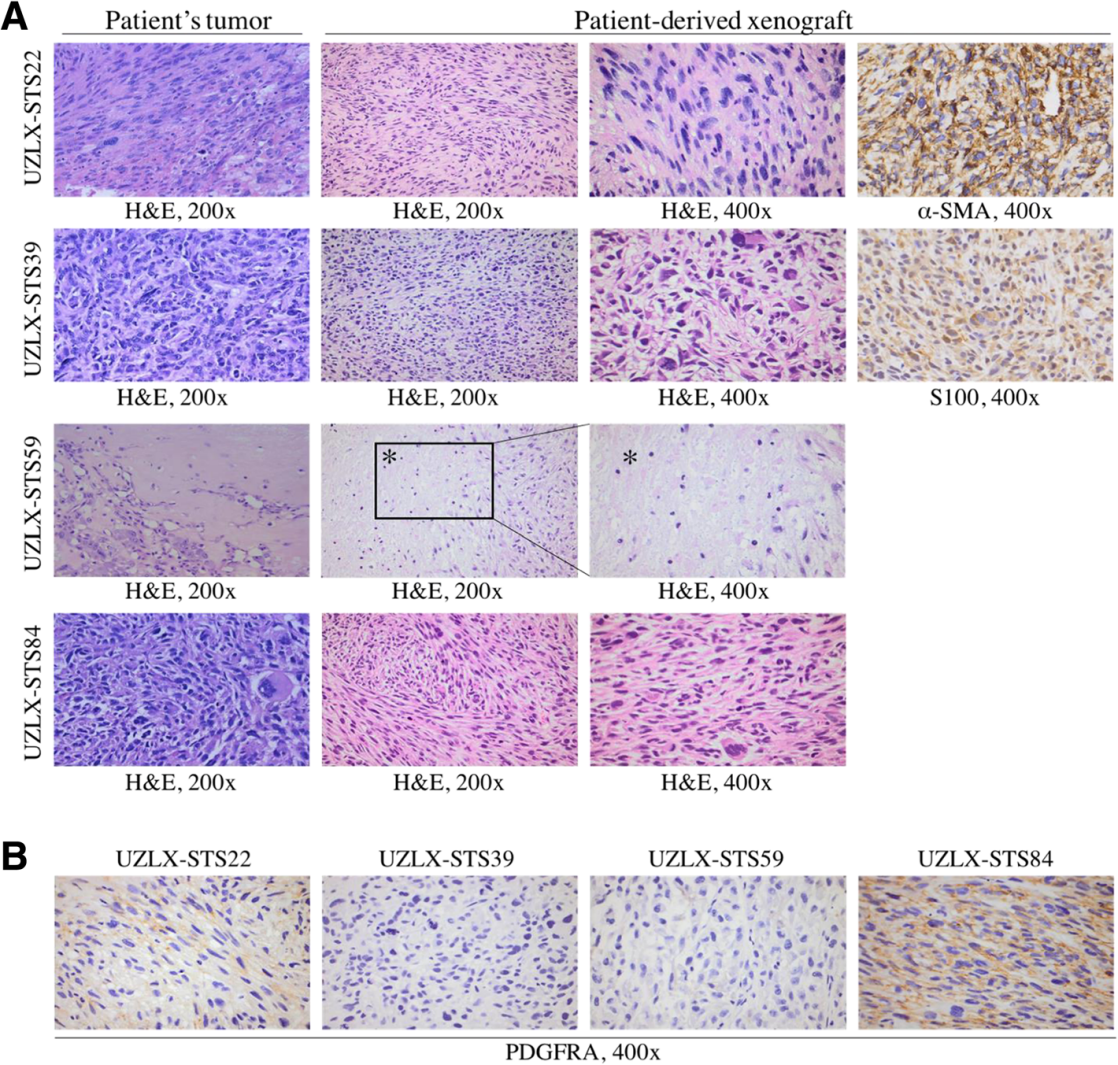
Characterization of patient-derived xenograft models. (**a**) Representative H&E and immunostainings of the original patients' tumors and the corresponding patient-derived xenograft models (passage used in the in vivo experiment). (**b**) PDGFRA immunostainings of control tumors of the different patient-derived xenograft models. 200x: 200-fold magnification; 400x: 400-fold magnification; α-SMA: alpha smooth muscle actin; H&E: hematoxylin and eosin; PDGFRA: platelet-derived growth factor receptor alpha; *: necrotic area in the UZLX-STS59 model

Original patients' tumors and corresponding PDX models shared the same histopathological features (Fig. 1a). Human origin of the xenografted tissue was confirmed by HLA-A positivity in all models (data not shown). Both the original patient’s tumor and ex-mouse UZLX-STS22p.18 LMS showed characteristic spindle cell morphology with diffuse α-SMA positivity. As for the UZLX-STS39 MPNST model, the original and xenografted sample (p.15) consisted of cells with a variable cellularity which showed immunopositivity for the neuronal marker S100. The original patient’s tumor of the UZLX-STS59 model was described as pleomorphic and predominantly necrotic, and those high-grade features were also present in the UZLX-STS59p.19 PDX model. The UZLX-STS84 original patient’s tissue and xenografted tissue (p.10) showed similar nuclear pleomorphism.

Initial PDGFRA immunostaining for model selection was assessed by Eli Lilly and Company on tissue slides from all PDX models available in the Laboratory of Experimental Oncology. Models UZLX-STS22p.11, UZLX-STS59p.14 and UZLX-STS84p.10 showed high intensity immunopositivity in 100% of the tumor cells, whereas UZLX-STS39p.9 showed positivity in 50% of tumor cells. Based on these findings, the abovementioned models were selected for subsequent in vivo experiments. However, when PDGFRA immunostaining was performed on the later passages used in the in vivo experiments, immunopositivity was only found in UZLX-STS22p.18 (weak) and UZLX-STS84p.10 (Fig. 1b).

### In vivo efficacy experiments

**Fig. 2:**
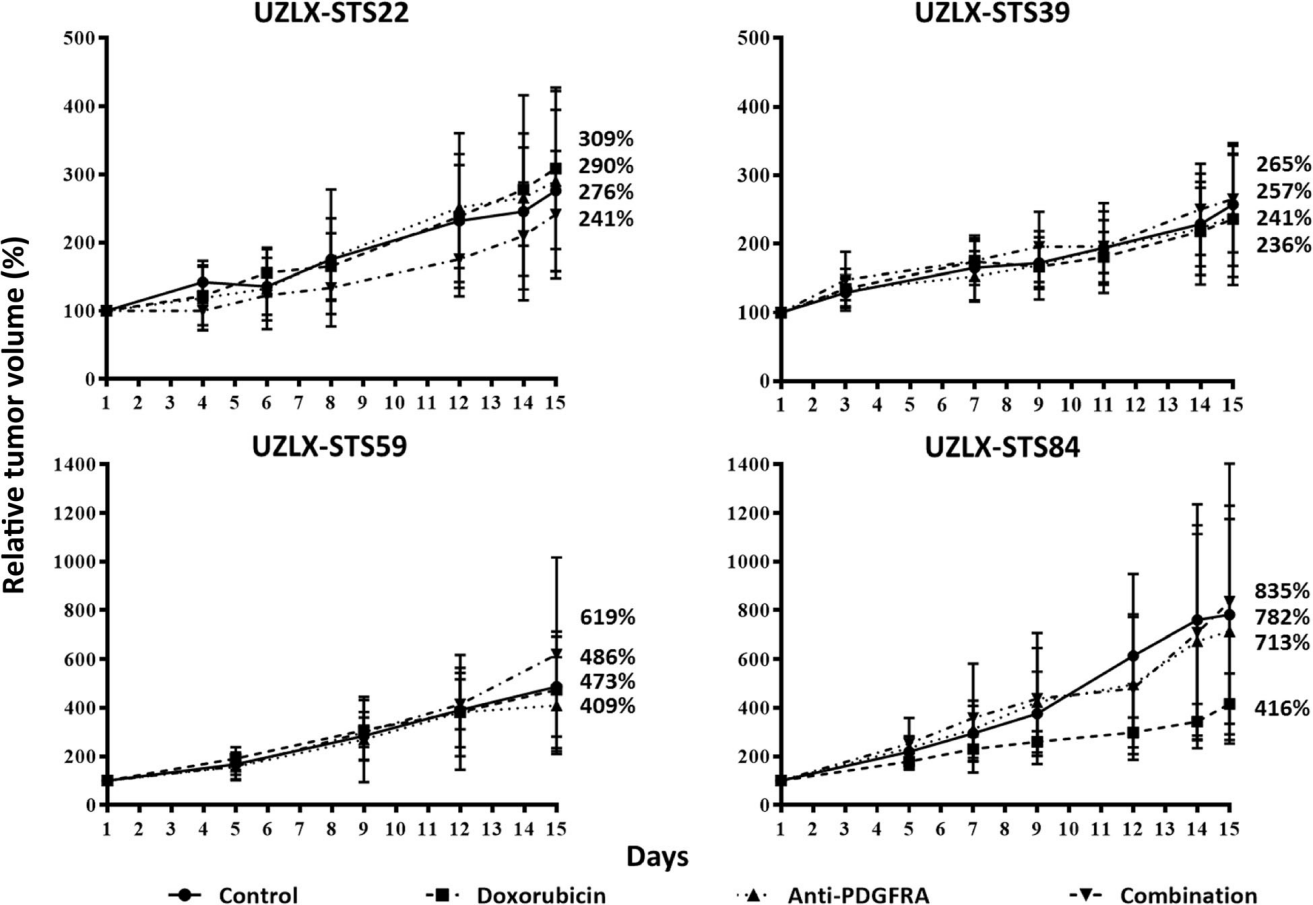
Tumor volume assessment during treatment. Tumor volume evolution in the UZLX-STS22 leiomyosarcoma, UZLX-STS39 malignant peripheral nerve sheath tumor, UZLX-STS59 myxofibrosarcoma and UZLX-STS84 undifferentiated pleomorphic sarcoma patient-derived xenograft model. Data are presented as relative tumor volume (%) compared to baseline. All data points are shown as mean ± standard deviation of at least seven tumors per treatment group. †: one UZLX-STS84-bearing mouse of the combination group sacrificed on day 13 due to a relative body weight of 85%, tumor volume before euthanasia included in the graph

Relative tumor volumes after the 2-week treatment period are shown in Fig. 2. Neither of the experimental treatments reduced the tumor growth in any of the PDX models tested (*P* > 0.05 compared to control, MWU). Although doxorubicin delayed tumor growth in the UZLX-STS84 PDX, the tumor volume at the end of the treatment was not significantly reduced compared to control (*P* = 0.15, doxorubicin vs. control, day 15, MWU).

Mean baseline body weight was 31.3 g (range 26–37 g). Overall, no significant body weight loss or other adverse events were observed in any of the treatment arms and the experimental compounds were well tolerated (Additional file 1: Figure S1). One UZLX-STS84-bearing mouse of the combination treatment arm was sacrificed on day 13 due to a body weight loss of 15%. Data from this drop-out animal were included in the graph of body weight and tumor volume (until day 13) but excluded from the statistical analysis of tumor volume and histopathological assessment, in order to only compare baseline samples vs. samples collected after the 2-week treatment period. All other animals/tumors were included in each analysis.

### Assessment of proliferation, apoptosis and microvessel density

**Fig. 3:**
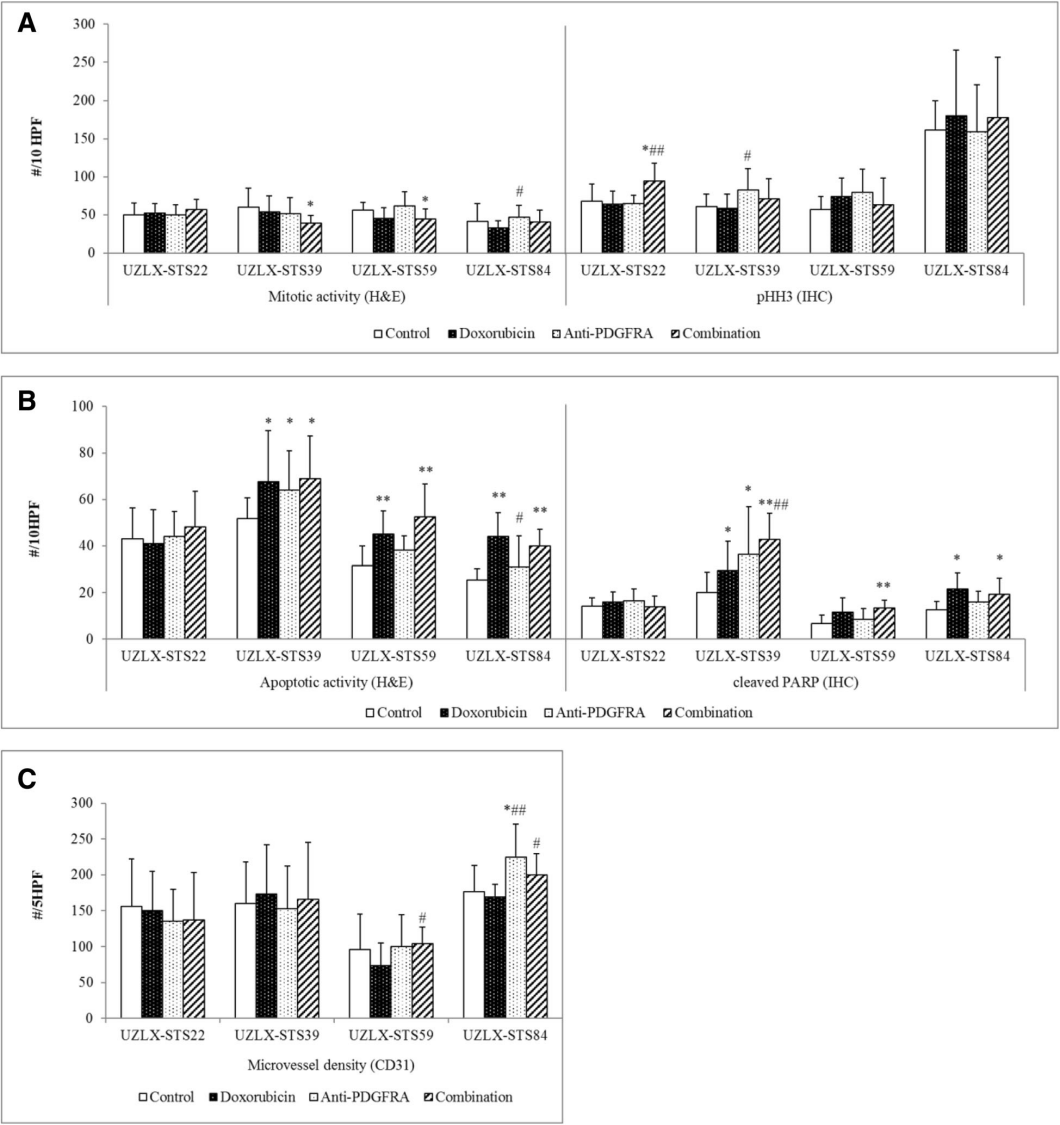
Assessment of proliferation, apoptosis and microvessel density. (**a**) Mitotic activity assessed by H&E staining and pHH3 immunostaining. (**b**) Apoptotic activity assessed by H&E staining and cleaved PARP immunostaining. (**c**) Microvessel density assessed by CD31 immunostaining. *p < 0.05, **p < 0.005 as compared to control; ^#^*p* < 0.05, ^##^*p* < 0.005 as compared to doxorubicin monotherapy

Mitotic and apoptotic activity in the tumors after the 2-week treatment period were assessed using H&E, pHH3 and cleaved PARP immunostaining. As compared to control, mitotic activity was not reduced by doxorubicin or anti-PDGFRA single treatment. In the PDX models UZLX-STS39 and -STS59 a significant reduction of proliferation compared to control was observed in the combination treatment arm, however, combining doxorubicin and anti-PDGFRA treatment did not lead to an additive anti-proliferative effect compared to the doxorubicin single agent treatment (Fig. 3a).

In the PDX models UZLX-STS39, -STS59 and -STS84 we observed an increase in apoptotic activity in the doxorubicin and combination treatment arms (*p* < 0.05 for UZLX-STS39, *p* < 0.005 for UZLX-STS59 and -STS84). The pro-apoptotic effect observed in the combination treatment arm was most likely attributable to the effect of doxorubicin, as no significant difference in apoptosis was observed between combination treatment and doxorubicin monotherapy. Furthermore, single agent anti-PDGFRA treatment did not increase the apoptotic activity in the xenografts (Fig. 3b).

Since in the anti-PDGFRA arms the treatment combined both an anti-human and -mouse antibody, it was also possible to assess the effects on the murine stromal compartment. A potential antiangiogenic effect of the experimental treatment was assessed by CD31 immunostaining. No significant reduction in tumor vascularization was observed between the control group and any of the experimental treatment arms, nor in the combination treatment arm compared to the doxorubicin single agent arm (Fig. 3c).

### PDGFRA pathway activation

**Fig. 4:**
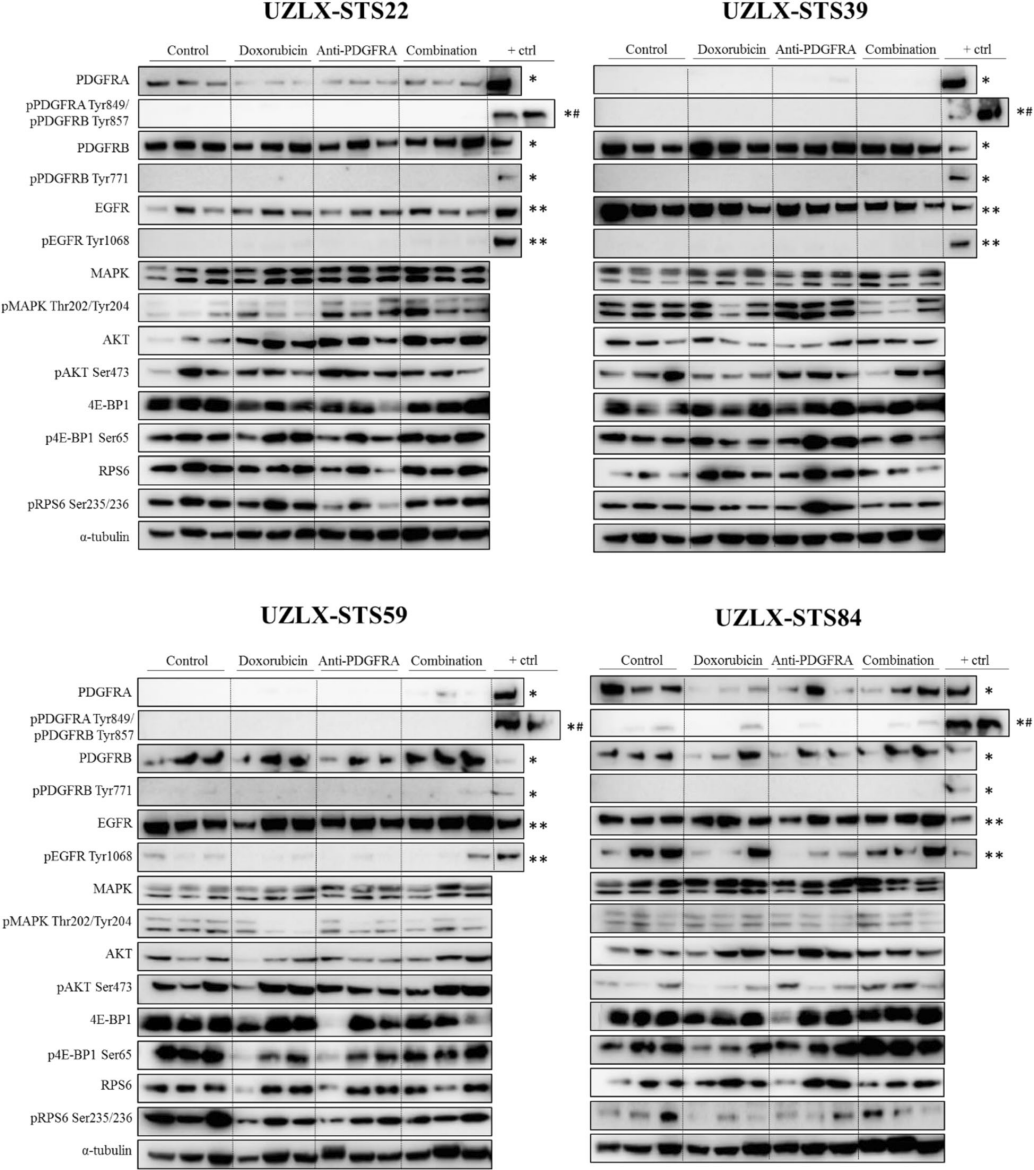
Western blot analysis of ex-mouse tumors. Western blot analysis of (p)PDGFRA/B, (p)EGFR and downstream MAPK and PI3K/AKT signaling pathways, performed on three different tumors per treatment group, collected after the 2-week treatment period. *MG-63 osteosarcoma cells stimulated with PDGF-BB, ^#^MG-63 osteosarcoma cells stimulated with PDGF-AA, **HEK cells

In accordance with the immunohistochemical findings, Western blot analysis showed PDGFRA expression in the control tumors of the UZLX-STS22 and -STS84 models, but not in UZLX-STS39 and -STS59. Additionally, PDGFRB and EGFR were consistently expressed in all models, but these receptor tyrosine kinases were not found to be activated, as they were not phosphorylated in control nor treated samples, except for EGFR in the UZLX-STS84 model (Fig. 4). Nevertheless, in the latter model anti-PDGFRA treatment did not lead to an inhibition of pEGFR. Neither of the experimental treatment arms markedly reduced the phosphorylation of any of the downstream signaling molecules MAPK, AKT, 4E-BP1 and RPS6.

## Discussion

Many chemotherapeutic agents have been investigated in the setting of advanced sarcoma, with only a handful of agents showing meaningful efficacy in unselected STS populations [5, 24, 25]. With doxorubicin remaining the standard first line therapy, prognosis for patients with advanced STS is still disappointingly poor. Hence, there is an important need for alternative treatment options beyond the traditional chemotherapeutic approach. In 2016, Tap et al. published the surprising results of a phase Ib/II randomized open label study in advanced STS, comparing doxorubicin monotherapy with doxorubicin and the anti-PDGFRA monoclonal antibody olaratumab [19]. Considering the remarkable OS benefit of 11.8 months observed in the study (median OS of 14.7 months in the doxorubicin group compared to 26.5 months in the combination group), FDA and EMA both granted accelerated approval for olaratumab in the setting of advanced STS. As the phase Ib/II study did not provide a good scientific rationale for the observed efficacy of olaratumab in combination with doxorubicin, we wanted to explore the effects of the drug in PDX models of relevant subtypes of STS enriched for PDGFRA expression.

We investigated the efficacy of olaratumab alone and in combination with doxorubicin in different STS subtypes, to gain further insight into how the drug works in this family of diseases.

Olaratumab as a single agent did not reduce tumor burden, did not impact mitotic or apoptotic activity and did not inhibit PDGFR signaling in any of the patient-derived models. We only observed an increase in apoptosis under anti-PDGFRA treatment in the UZLX-STS39 MPNST model. Under doxorubicin no relevant antitumor activity in terms of tumor volume, proliferative activity or microvessel density was observed, but an increase in apoptotic activity in all PDX except UZLX-STS22 was found, illustrating the high chemotherapy resistance of the selected models. The lack of efficacy of doxorubicin in these high-grade PDX models is not unexpected, as response rates in the clinic are only around 15% [4]. In this context it is worth mentioning that two of the four donor patients were exposed to doxorubicin after providing tissue for xenografting, and none of them experienced clinical benefit. In our study, adding olaratumab to doxorubicin did not result in an increased antitumor effect in the patient-derived models.

There could be several explanations for the lack of efficacy of olaratumab in our STS PDX models. Firstly, we did only observe expression of PDGFRA in two out of the four PDX models used in the experiments, in contrast to the initial immunohistochemical analysis for model selection performed by Eli Lilly and Company on earlier passages of the models, in which all four models showed PDGFRA immunopositivity. Retrospectively, we repeated the analysis performed on the earlier passages, and results obtained in the Laboratory of Experimental Oncology were discordant with the results reported by Eli Lilly and Company. In our hands, immunohistochemical profiles of the earlier passages and the passages used in the in vivo experiment were identical, showing only PDGFRA-immunopositivity in UZLX-STS22 and -STS84. Furthermore, our results were confirmed by Western immunoblotting, including adequate positive controls to exclude false negative results. This implicates that the expression of PDGFRA in UZLX-STS39 and -STS59 was not lost but was already absent in the passages used for model selection. However, the two PDGFRA-negative models did serve as internal negative controls in the experimental set-up. Nevertheless, there was no correlation between the presence of PDGFRA and response to olaratumab. Of note, none of the PDGFRA-positive models revealed activation of the receptor, as assessed by the presence of its phosphorylation. As a consequence, olaratumab could not inhibit the activation of PDGFRA and his downstream effector signaling molecules such as MAPK, AKT, 4E-BP1 and RPS6. Of note, PDGFRA expression was seen in around one third of STS in the clinical study of Tap et al., but PDGFRA expression did not correlate with treatment outcome in terms of OS or PFS [19]. Tumor tissue samples were examined for PDGFRA expression by IHC, but activation of PDGFRA was not assessed in the clinical study. Potentially, the PDGFRA-positive tumors did not express the activated form of the receptor, as was the case in our in vivo study. Due to the lack of activation of PDGFRA in the PDGFRA-positive models, this study could not fully assess the potential of olaratumab in PDGFRA-driven oncogenicity. Potentially, PDGFRA activation might be the main driver of tumorigenesis in a small portion of STS, and olaratumab could have an antitumoral effect in this subgroup of patients. Research models and experimental designs for further preclinical work should be selected not only on PDGFRA expression, but on the presence of activated PDGFRA as a driver of tumor growth. Such an experimental set-up would allow to fully assess and potentially demonstrate the non-efficacy of olaratumab in PDGFRA-driven sarcomagenesis.

Hypothetically, off-target effects of olaratumab might explain the discrepancy between the surprising survival impact of the drug in the earlier clinical trial and the lack of a correlation with PDGFRA expression. In contrast with these results, Lowery et al. recently showed a positive correlation between the response to olaratumab and PDGFRA expression in STS xenografts [18]. The four models described in these experiments showed high expression of PDGFRA and two out of four models showed absence of PDGFRB. Furthermore, in vitro evaluation of several cell lines showed negative correlation with PDGRB expression, potentially indicating the importance of exclusive PDGFRA expression. However, in-depth knowledge about the presence and role of PDGFRA or other receptor tyrosine kinases in sarcoma cells or tumor microenvironment is still scarce.

Wallin et al. described increased phosphorylation of AKT upon doxorubicin treatment in breast and ovarian cancer cell lines, inducing a synergistic antitumor effect when combining doxorubicin with an agent targeting the PI3K/AKT pathway [26]. However, doxorubicin did not increase phosphorylation of AKT in our PDX models, potentially masking the inhibitory effect of olaratumab on the PI3K/AKT pathway in the combination arm.

PDX mice might not be optimal research models to explore the efficacy of agents affecting the tumor microenvironment, while olaratumab may hypothetically act via the surrounding stroma. It has been consistently shown that the human tumor stroma in PDX models is replaced by murine stroma after 3–5 passages [22]. Tumor stroma in our PDX models was thus in essence murine. However, PDGF produced by these non-tumorigenic murine stromal cells may still affect tumor stroma recruitment and growth. This paracrine stimulation by PDGF also acts on murine pericytes around blood vessels and murine (myo)fibroblasts in the stroma, resulting in increased interstitial fluid pressure, being a well-described obstacle for chemotherapy drug delivery [13, 15]. This effect was taken into account in our study design, by treating mice not only with olaratumab, but with an anti-PDGFRA mixture consisting of both human and murine anti-PDGFRA antibody. Nevertheless, the role of infiltrating inflammatory cells in promoting tumor growth cannot be fully assessed in the partially immunodeficient mouse strain used in this study. Moreover, a hypothetical interaction of the anti-PDGFRA antibody with immunocompetent cells in tumor and/or stroma can also not be assessed in these immunodeficient animal models.

The results of this study are to be confirmed by larger follow-up studies in models of different STS subtypes and preferably also in more advanced translational models such as humanized PDX models. Humanized PDX are generated by co-engrafting human tumor tissue and human immune cells, mostly through transplantation of human hematopoietic stem cells, resulting in immune reconstitution in mice [27]. These models should allow to investigate the role of tumor associated immune cells with regard to tumor growth and survival. More comprehensive preclinical research involving a variety of different experimental approaches is required to fully assess and perhaps confirm the failure of anti-PDGFRA targeting in the field of soft tissue sarcoma.

Another consideration to make when evaluating the discrepancy between these negative preclinical results and the promising results observed in the phase Ib/II clinical trial, are the potential methodological limitations of the actual clinical study that stimulated this in vivo work. The phase II portion of the trial, randomizing 133 patients between doxorubicin monotherapy vs. doxorubicin plus olaratumab, was open label, had no blinding and was not placebo-controlled. Further variations in disease biology between both treatment groups and different post-protocol local and/or systemic treatments might have impacted the results. Of note, many patients in the doxorubicin arm received single agent olaratumab as a second line treatment, an agent that – as the study demonstrated – has only limited non-significant effect on PFS when combined with doxorubicin in the first line setting, and a drug that does not seem to have relevant single agent activity in our PDX mice. Patients in the combination arm of the clinical trial were more likely to receive gemcitabine/docetaxel, pazopanib or trabectedin, all agents which are known to be active second line treatments, associated with a significant prolongation in PFS in randomized clinical studies [6, 7, 25]. The median number of doxorubicin cycles administered in the study of Tap et al. in the combination and doxorubicin group were seven and four cycles, respectively. Accordingly, the median cumulative dose of doxorubicin administered was 488 mg/m^2^ in the combination arm vs. 300 mg/m^2^ in the single agent doxorubicin arm [19]. As a consequence, the higher cumulative anthracycline exposure in the combination arm could have contributed to the increased efficacy observed when combining doxorubicin and olaratumab in the phase Ib/II clinical trial. Of note, no correlation between PDGFRA expression and treatment outcome was found.

Interestingly, our negative results showing the lack of activity of olaratumab in different PDX of sarcoma, are in line with the very recent release reporting that the large, placebo-controlled ANNOUNCE phase III trial (www.clinicaltrials.gov NCT02451943) did not meet the OS primary endpoint in STS patients, as there was no difference in survival between the doxorubicin single treatment arm and the combination arm with doxorubicin and olaratumab (Eli Lilly and Company, press release January 2019).

## Conclusions

We were not able to demonstrate efficacy of olaratumab in patient-derived STS xenografts. We did not demonstrate significant antitumor effect of anti-PDGFRA treatment, neither alone nor in combination with doxorubicin. Other translational studies are currently being conducted in an attempt to identify the exact role of PDGFRA in sarcomagenesis and the mechanism of action and in vivo effects of olaratumab. Our data support the negative findings of the phase III ANNOUNCE trial and confirm the reliability of patient-derived xenograft models in translational cancer research.

## Additional files

Additional file 1: **Figure S1.** Body weight assessment during treatment. Body weight evolution in the UZLX-STS22 leiomyosarcoma PDX, UZLX-STS39 malignant peripheral nerve sheath tumor PDX, UZLX-STS59 myxofibrosarcoma PDX and UZLX-STS84 undifferentiated pleomorphic sarcoma PDX model. Data are presented as relative body weight (%) compared to baseline. All data points are shown as mean ± standard deviation of at least five mice per treatment group. †: one UZLX-STS84-bearing mouse of the combination group sacrificed on day 13 due to a relative body weight of 85%. (PDF 187 kb)

Additional file 2: **Table S1.** Detailed description of the number of mice/tumors included in the in vivo experiments. (DOCX 18 kb)

## Data Availability

The datasets used and/or analyzed during the current study are available from the corresponding author on reasonable request.

## Abbreviations

1E10: Mouse anti-PDGFRA
4E-BP1: Eukaryotic translation initiation factor 4E-binding protein 1
AIDA: Advanced Image Data Analyzer
BIW: Twice a week
DAB: Diaminobenzidine
EGFR: Epidermal growth factor receptor
EMA: European Medicines Agency
FDA: Food and Drug Administration
H&E: Hematoxylin and eosin
HEK293: Human embryonic kidney 293 cells
HLA-A: Human leucocyte antigen A
HPF: High power field
IgG1: Immunoglobulin G subclass 1
IHC: Immunohistochemistry
LMS: Leiomyosarcoma
MAPK: Mitogen-activated protein kinase
MPNST: Malignant peripheral nerve sheath tumor
MVD: Microvessel density
MWU: Mann-Whitney U test
ORR: Objective response rates
OS: Overall survival
p4E-BP1: Phospho-4E-BP1 Ser65
pAKT: phospho-AKT Ser473
PARP: Poly (ADP-ribose) polymerase
PDGF: Platelet-derived growth factor
PDGFRA: Platelet-derived growth factor receptor alpha
PDGFRB: Platelet-derived growth factor receptor beta
PDX: Patient-derived xenografts
pEGFR: phospho-EGFR Tyr1068
PFS: Progression-free survival
pHH3: Phospho-histone H3
PI3K: Phosphoinositide 3-kinase
pMAPK: Phospho-MAPK Thr202/Tyr204
pPDGFRA: phospho-PDGFRA Tyr 849
pPDGFRB: phospho-PDGFRB Tyr 857
pRPS6: Phospho-RPS6 Ser235/236
PVDF: Polyvinylidene difluoride
QW: Once a week
RPS6: Ribosomal protein S6
STS: Soft tissue sarcoma
TMA: Tissue microarray
UPS: Undifferentiated pleomorphic sarcoma
VEGF: Vascular endothelial growth factor
WHO: World Health Organization
WMP: Wilcoxon matched pairs test
α-SMA: Alpha-smooth muscle actin
