# Supplementary figures and images for: Assessment of the platelet-derived growth factor receptor alpha antibody olaratumab in a panel of patient-derived soft tissue sarcoma xenografts
Source: BMC Cancer. 2019 Jul 22;19:724. doi: 10.1186/s12885-019-5872-1 (PMC6647161; doi:10.1186/s12885-019-5872-1)

Cornillie et al. Supplementary Figure 1

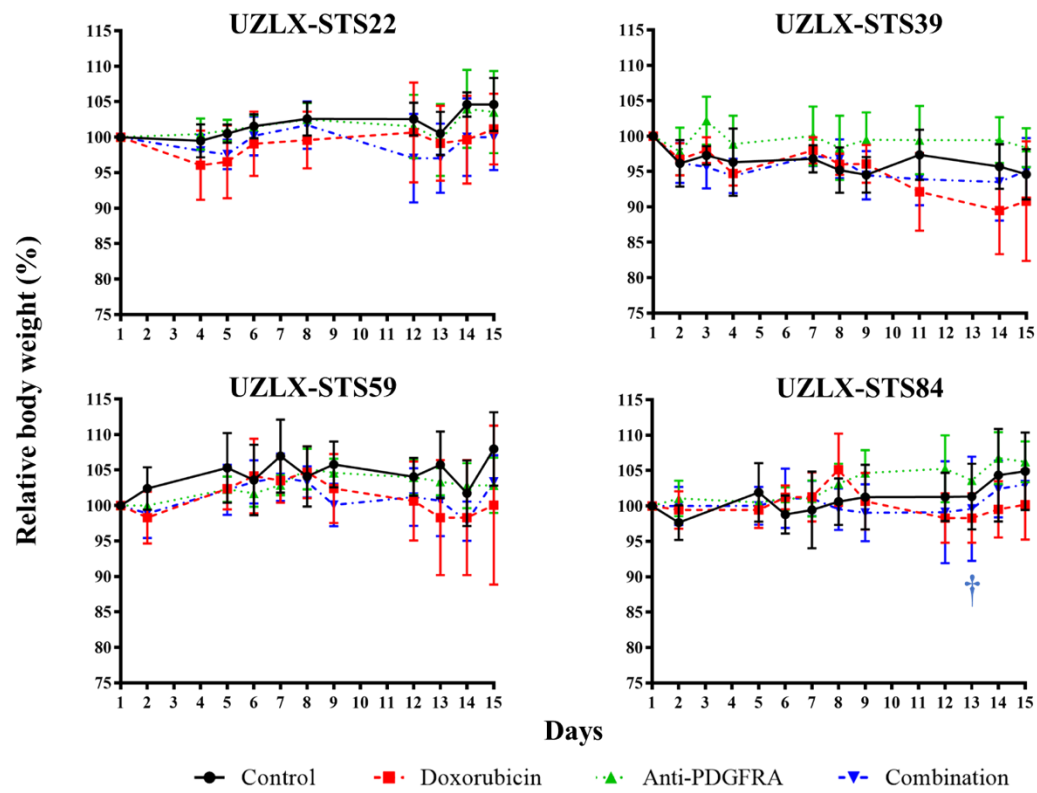

Supplement: Supplementary file 1 — Figure S1. Body weight assessment during treatment. Body weight evolution in the UZLX-STS22 leiomyosarcoma PDX, UZLX-STS39 malignant peripheral nerve sheath tumor PDX, UZLX-STS59 myxofibrosarcoma PDX and UZLX-STS84 undifferentiated pleomorphic sarcoma PDX model. Data are presented as relative body weight (%) compared to baseline. All data points are shown as mean ± standard deviation of at least five mice per treatment group. †: one UZLX-STS84-bearing mouse of the combination group sacrificed on day 13 due to a relative body weight of 85%. (PDF 187 kb) [file 12885_2019_5872_MOESM1_ESM.pdf]
