# Supplementary material for: Assessment of the platelet-derived growth factor receptor alpha antibody olaratumab in a panel of patient-derived soft tissue sarcoma xenografts
Source: BMC Cancer. 2019 Jul 22;19:724. doi: 10.1186/s12885-019-5872-1 (PMC6647161; doi:10.1186/s12885-019-5872-1)
Supplement: Supplementary file 2 — Table S1. Detailed description of the number of mice/tumors included in the in vivo experiments. (DOCX 18 kb) [file 12885_2019_5872_MOESM2_ESM.docx]

| **^Xenograft model (passage)^** | **^Control^** | **^Doxorubicin^** | **^Anti-PDGFRA^** | **^Combination^** |
| --- | --- | --- | --- | --- |
| ^UZLX-STS22(p18)^ | ^6 (12)^ | ^6 (10)^ | ^6 (12)^ | ^6 (12)^ |
| ^UZLX-STS39(p15)^ | ^6 (11)^ | ^5 (10)^ | ^6 (12)^ | ^6 (12)^ |
| ^UZLX-STS59(p19)^ | ^6 (11)^ | ^6 (12)^ | ^6 (12)^ | ^6 (12)^ |
| ^UZLX-STS84(p10)^ | ^5 (7)^ | ^5 (8)^ | ^6 (9)^ | ^7 (12)^ |

number of mice entered in the experiment; between brackets: number of tumors entered in the experiment; PDGFRA: platelet-derived growth factor receptor alpha
